# Clinical implementation of MR‐guided vaginal cylinder brachytherapy

**DOI:** 10.1120/jacmp.v16i6.5460

**Published:** 2015-11-08

**Authors:** Amir M. Owrangi, Shruti Jolly, James M. Balter, Yue Cao, Katherine E. Maturen, Lisa Young, Tong Zhu, Joann I. Prisciandaro

**Affiliations:** ^1^ Department of Radiation Oncology University of Michigan Ann Arbor MI; ^2^ Department of Radiology University of Michigan Ann Arbor MI USA

**Keywords:** brachytherapy, MRI, applicator reconstruction

## Abstract

We present an institutional experience on the clinical implementation of magnetic resonance (MR)‐guided vaginal brachytherapy using commercially available solid applicator models. To test the fidelity of solid applicator models to digitize vaginal cylinder applicators, three datasets were evaluated. The first included 15 patients who were simulated with CT alone. Next, a water phantom was used to evaluate vaginal cylinders ranging from 20 to 35 mm in diameter with CT and MR. Finally, three patients undergoing HDR brachytherapy with vaginal cylinders that were simulated with both CT and MR were evaluated. In these assessments, the solid applicator models were aligned based on the outline of the applicators on the corresponding volumetric image, and deviations between the central source positions defined based on X‐ray markers (on CT) and solid applicator models (on CT and MR), and the percent dose difference between select reference points were calculated. The mean central source deviation defined based on X‐ray markers (on CT) and solid applicator models (on CT and MR) for the 15‐patient cohort, the phantom, and the 3‐patient cohort is 0.6 mm, 0.6 mm, and 1.2 mm, respectively. The average absolute percent dose difference for the bladder, rectum, prescription, and inferior reference points were 2.2%, 2.3%, 2.2%, and 2.4%, respectively, for the 15 patient cohort. For the phantom study, the average, absolute percent dose difference for the prescription and inferior reference points are 2.0% and 2.1% for the CT, 2.3% and 2.2% for the T1W, and 2.8% and 3.0% for the T2W images. For the three patient cohort, the average absolute percent dose difference for the bladder, rectum, prescription, and inferior reference points are 2.9%, 2.6%, 3.0%, and 4.2% for the CT, 6.5%, 1.6%, 2.5%, and 4.7% for the T1W, and 6.0%, 7.4%, 2.6, and 2.0% for the T2W images. Based on the current study, aligning the applicator model to MR images provides a practical, efficient approach to perform MR‐based brachytherapy planning.

PACS numbers: 87.53.Jw, 87.61.Tg

## I. INTRODUCTION

Endometrial carcinoma is the most prevalent gynecological cancer in the United States and accounts for 6% of all cancers in females.^(1)^ Most cancers are detected at an early stage and the long‐term prognosis is excellent. Currently, postoperative vaginal brachytherapy, with or without external beam radiation therapy, is one of the main components of endometrial cancer treatment. Based on the Postoperative Radiation Therapy for Endometrial Carcinoma‐2 (PORTEC‐2) study of early‐stage postoperative endometrial cancer patients, similar results in preventing distal metastasis and local recurrences were observed with patients receiving external beam radiation therapy as with brachytherapy alone.^(2)^ However, it has been shown that patients who received brachytherapy alone develop less gastrointestinal complications and, in general, vaginal cylinder brachytherapy may provide better quality of life outcomes, in comparison to external beam radiation therapy.^3^, ^4^, ^5^

Broadly speaking, the aim of radiotherapy is to maximize the dose received by a specific region of interest while minimizing the dose to the surrounding normal structures. To achieve this end with brachytherapy, the dwell positions and times of the radioactive source(s) needs to be optimized. Traditionally, treatment planning for vaginal brachytherapy has relied on radiographs and the presence of an X‐ray marker for source channel localization and digitization (known as applicator reconstruction). However, radiographs do not provide sufficient soft‐tissue contrast to visualize the targeted tissue due to limited differences in attenuation between the tumor and surrounding normal tissue. As a result, traditional treatment plans have been designed to deliver the prescribed dose to a specific point relative to the applicator geometry to which anatomic significance is attached (e.g., for cervical cancer, dose has traditionally been prescribed to Point A^(6)^).

Over the past decade, volumetric imaging has largely replaced 2D imaging for support of brachytherapy treatment planning.^(7)^ Unlike radiographs, volumetric images support some visualization of tumors and adjacent normal soft tissues. The Groupe Européen de Curiethérapie — European Society for Radiotherapy & Oncology (GEC‐ESTRO) has recognized the importance of volumetric imaging, in particular magnetic resonance (MR) imaging (MRI), and 3D treatment planning for cervical cancer, and has published a series of recommendations^8^, ^9^, ^10^, ^11^ to assist in the standardization of volume and MR‐based treatment planning for cervical cancer. However, similar recommendations are not currently available for endometrial cancer. Clinically, the use of MRI in the planning of vaginal brachytherapy enables detailed evaluation of the vaginal cuff, in addition to assessing dose delivered to clinically relevant anatomic structures of the pelvis, that may be important for sexual and/or pelvic floor dysfunction.

With the introduction of MRI simulators in radiotherapy departments, there is a need for further guidance on the commissioning and clinical implementation of MR‐guided brachytherapy. This manuscript serves to present an institutional experience on applicator and imaging commissioning, as well as clinical implementation of MR‐guided vaginal brachytherapy.

## II. MATERIALS AND METHODS

To clinically commission the use of MR‐guided brachytherapy for vaginal cylinders, commercially available standard and segmented vaginal cylinders (Varian Medical Systems, Palo Alto, CA) were scanned using computed tomography (CT) and MR imaging systems. Computed tomography scans were acquired using a 16‐slice CT scanner (Royal Philips Electronics, Eindhoven, The Netherlands) with 1 mm slice thickness. MR scans were performed using a 3T wide‐bore MRI simulator (Skyra, Siemens Medical Systems, Erlangen, Germany). The following MRI sequences were used: 3D T2‐weighted (SPACE) coronal (FOV: 320×320×176 mm, voxel size: 0.94×0.94×1mm, TR/TE: 1700/88 ms), and 3D T1‐weighted (MPRAGE) coronal (FOV: 300×300×166.4 mm, voxel size: 1.17×1.17×1.3 mm, TR/TE/TI: 1900/2.35/900 ms, flip angle: 9°). The MRI simulator is outfitted with a laser marking system (LAP Laser, Luneburg, Germany) and detachable couch.^(12)^ The couch supports imaging, as well as treatment of brachytherapy patients, eliminating the need to transfer patients, thus reducing the risk of inadvertently modifying the local geometry of the applicator and surrounding tissues.

Three datasets were analyzed to aid in the commissioning of MR‐guided vaginal cylinder brachytherapy. The first dataset was from 15 patients who underwent vaginal brachytherapy and were simulated with CT alone. The second dataset was a series of phantom scans of the standard and segmented vaginal cylinders ranging in diameter from 20 mm to 35 mm. In this assessment, all applicators were simulated in a gadolinium‐doped water phantom using CT and MR imaging. The third dataset came from three patients undergoing HDR brachytherapy with vaginal cylinders who were simulated with both CT and MR. The *in vivo* scans were acquired with patients positioned supine with their legs straight.

**Figure 1:**
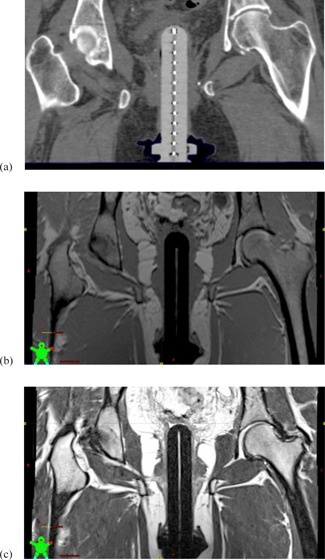
Coronal view of a patient with a 26 mm standard vaginal cylinder on (a) CT, (b) 3D T1W (MPRAGE) MR, and (c) 3D T2W (SPC) MR. To assist with the visualization of the central source channel, the appropriate marker (X‐ray for CT and contrast filled for MR) was inserted in the applicator prior to simulation.

Prior to each of the treatment planning simulations, a marker was inserted into the center of the applicator to assist with the visualization and digitization of the applicator tip and source path in the treatment planning software. For CT simulation, a commercial X‐ray marker was utilized (Varian Medical System, Palo Alto, CA). Figure 1(a) shows a sample CT scan of a 26 mm diameter standard vaginal cylinder applicator with an X‐ray marker in place. For MR simulation, an in‐house MR marker was made using a thin (1.168 mm outer diameter), hollow nylon tube (Best Medical International, Springfield, VA) filled with gadolinium‐doped water (T1 contrast) or either water or 0.25% Agarose Gel (T2 contrast), then sealed. Agarose gel was added to increase the viscosity of water and reduce the mobility of the fluid in the tube. Once the tubes were filled, several different techniques were tested to seal the catheters. Figures 1(b) and 1(c) show representative MR images acquired with 3D T1‐weighted (T1W) and T2‐weighted imaging sequences as detailed above.

For treatment planning purposes, the CT and MR images were imported into a commercial treatment planning system (BrachyVision 8.11, Varian Medical Systems) which allows the user to identify the tip of the applicator, the position of the applicator/potential source positions, and the relevant patient anatomy. All clinical treatment plans were generated using the CT images. The proximal 100 mm of the applicator was digitized in the conventional manner based on the location of the X‐ray marker. Since the desired treatment length was the proximal 40 mm of the applicator, to ensure adequate dose coverage, the first 50 mm of the applicator (10 dwell positions, with a 5 mm step size) contained active dwell positions. Once the applicator channel was digitized, reference points were added to monitor the dose to a nominal prescription point (applicator surface at one‐half of the total treatment length as defined by the authorized user (AU) physician), an inferior reference point (applicator surface at a distance equivalent to the total treatment length from the apex of the vaginal cylinder), and the ICRU 38‐defined^(6)^ bladder and rectum points (only for clinical plans). Treatment plans for the phantom scans were generated to deliver a total of 12 Gy over 2 fractions to the prescription point. The clinical treatment plans for the three‐patient cohort were generated in a similar manner. However, since the initial prescription point for the 15‐patient cohort varied between surface and a depth of 5 mm due to a change in clinical practice, plans that were prescribed to a depth of 5 mm were retrospectively renormalized to deliver 12 Gy over 2 fractions to the surface of the vaginal cylinder.

In a retrospective analysis, applicator digitization on MR images using the in‐house MR markers were evaluated by registering the T1W and T2W MR images to the CT images based on visually aligning the images to the perimeter of the vaginal applicator. The registration was evaluated in the para‐axial, paracoronal, and parasagittal planes. Due to possible movement between scans, as well as possible systematic differences in the localization of markers from the MR scans acquired with different scanning sequences, the T1W and T2W images were separately aligned to the corresponding CT images.

To validate the commercially available solid applicator models in the treatment planning system (BrachyVision 8.11), we examined the accuracy of applicator reconstruction in CT for phantom and *in vivo* scans by comparing the digitization with our conventional technique, utilizing X‐ray markers. The library consists of a series of applicator models designed and made available by the vendor (Varian Medical Systems) for a number of commercial brachytherapy applicators, including vaginal cylinders. The applicator models were derived from the original computer‐aided design (CAD) drawings of the applicators (D. Harrington, Varian Medical Systems, personal communication, April 13, 2015). To test the fidelity of this digitization technique for the 15‐patient cohort and the phantom datasets, the solid applicator models were aligned based on the outline of the applicators on the corresponding volumetric images. The source position coordinates, as well as the calculated doses to the prescription and inferior points, were extracted and compared with those values determined based on the digitization using the X‐ray marker (our conventional applicator reconstruction technique).

Once validated, the solid applicator model was evaluated for applicator reconstruction using MR images. The accuracy of application reconstruction in MR images was assessed in a gadolinium‐doped water phantom study for standard and segmented vaginal cylinders. Similar to the validation study, applicator reconstruction based on the X‐ray marker in CT was considered our gold standard. Thus, to evaluate the source position accuracy, the central source position defined based on the solid applicator model in MR was compared to that defined by the X‐ray marker on CT.

To demonstrate the feasibility of using the solid applicator approach *in vivo* for MR‐based applicator digitization, MR and CT images were acquired for a three‐patient cohort undergoing vaginal cylinder HDR brachytherapy treatments. This analysis was limited to three patients due to limited staff and equipment resources, as well as patient discomfort and inconvenience.

The central source position defined by the solid applicator model on MR was compared to the coordinates defined using the X‐ray marker on CT. Additionally, the calculated dose to reference points of interests was also compared between the applicator reconstruction techniques.

## III. RESULTS

A summary of the distributions of central source positions defined by the conventional applicator reconstruction method versus the CT‐aligned solid applicator model for the 15‐patient cohort is presented in Table 1. The mean source position deviation across the 15 patients is 0.6 mm and the average absolute percent dose difference for the bladder, rectum, prescription, and inferior reference points are 2.2%, 2.3%, 2.2%, and 2.4%, respectively.

**Figure 2:**
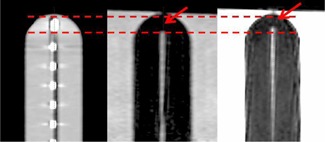
A paracoronal view of the CT (left), T1W (middle), and T2W (right) images of a 30 mm standard vaginal applicator with the appropriate CT or MR marker. In contrast to the CT, the MR markers for both the T1W and T2W images were displaced from the tip of the applicator, suggesting the markers were compromised.

Figure 2 shows sample CT and MR (T1W and T2W) images of a 30 mm standard vaginal cylinder in a gadolinium‐doped water phantom in paracoronal planes relative to the applicator. The vaginal applicator appears hypo‐intense on both MR image sets, and the source path is visible when an MR marker with the appropriate contrast agent was placed in the applicator prior to simulation. However, due to difficulties obtaining a water‐tight seal, this marker could not be made reproducibly, resulting in displacements of the visualized applicator tip. Similar results were observed clinically for the three‐patient cohort and, as a result, we discontinued the use of the MR marker in favor of the solid applicator models for applicator digitization.

**Table 1:** Magnitude of source displacement for the central source position and percent dose difference at reference points when applicator reconstruction based on the solid applicator model is compared to reconstruction based on X‐ray markers over the range of 10 dwell positions for 15 different patients simulated with CT alone

*Subject Number*	*Applicator Type*	*Min (mm)*	*Max (mm)*	*Mean (mm)*	*SD (mm)*	*% Dose Difference Prescription Point*	*% Dose Difference Inferior Reference Point*	*% Dose Difference Bladder*	*% Dose Difference Rectum*
1	Standard 3.0 cm	0.8	1.0	0.9	0.1	4.6	3.2	1.2	−0.6
2	Standard 2.6 cm	0.1	0.5	0.3	0.1	−0.8	−0.2	−0.5	−0.1
3	Standard 3.0 cm	0.9	1.0	1.0	0.0	1.5	−0.4	−4.4	5.3
4	Standard 3.5 cm	0.4	0.5	0.5	0.0	−1.1	0.1	−0.6	−1.5
5	Standard 2.0 cm	0.4	0.6	0.5	0.1	−6.3	−7.2	N/A	3.5
6	Standard 3.0 cm	0.6	0.8	0.7	0.1	−2.8	−3.1	−2.7	2.7
7	Standard 3.0 cm	0.6	0.8	0.7	0.1	−2.1	−0.6	−2.9	1.9
8	Segmented 2.6 cm	0.5	0.9	0.7	0.1	1.0	−0.3	N/A	−2.0
9	Standard 3.5 cm	0.3	0.5	0.4	0.0	0.9	−8.5	1.4	−1.7
10	Segmented 3.0 cm	0.5	0.7	0.6	0.0	−1.4	−4.9	−2.6	2.6
11	Standard 3.0 cm	0.4	0.6	0.5	0.1	1.3	0.5	2.1	−1.3
12	Standard 2.6 cm	0.6	0.9	0.8	0.1	−2.3	−2.1	4.0	−0.8
13	Segmented 2.6 cm	0.1	0.4	0.3	0.1	−2.7	−1.5	0.8	−3.6
14	Segmented 3.5 cm	0.4	0.8	0.6	0.1	−1.8	0.0	2.9	−3.7
15	Segmented 2.6 cm	0.6	0.7	0.6	0.0	2.6	3.5	2.7	−3.4

**Figure 3:**
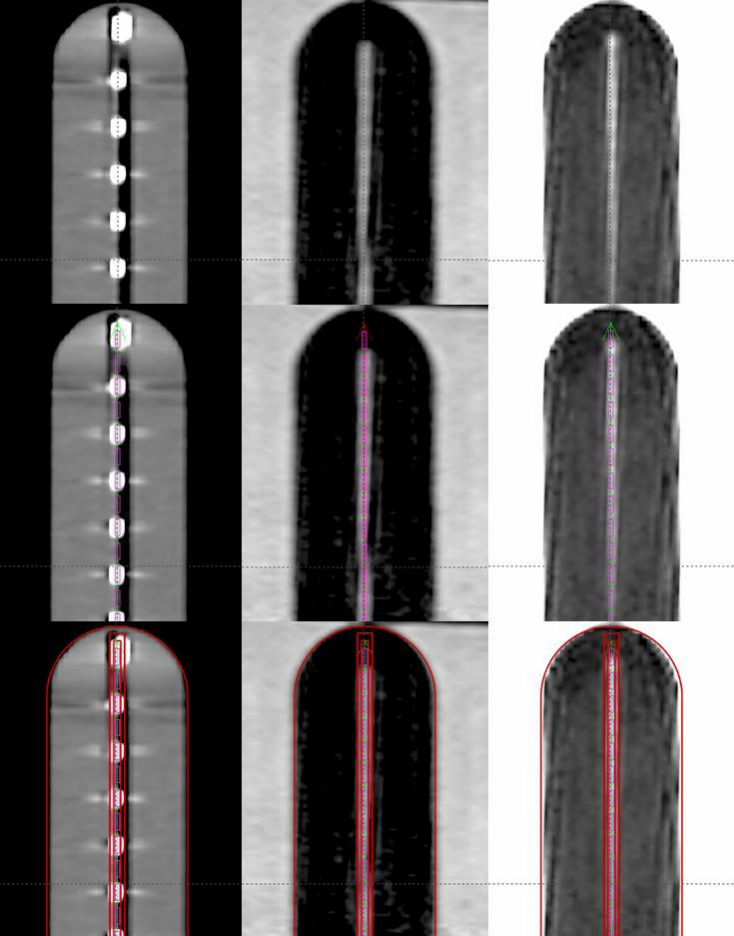
(Top row) Paracoronal view of the CT (left), T1W (middle), and T2W (right) images of the 30 mm standard vaginal cylinder with the appropriate marker in place. (Middle row) Applicator reconstruction using X‐ray markers on CT and overlaid on the MR images after the MR images were registered with the CT. (Bottom row) Applicator reconstruction using the solid applicator model aligned independently in each imaging modality.

Figure 3 shows sample CT, T1W and T2W MR images of a 30 mm standard vaginal cylinder in a gadolinium‐doped water phantom in paracoronal planes relative to the applicator with an overlay of the solid applicator model (in red) with the appropriate applicator diameter. A summary of the distributions of differences in central source positions defined based on the X‐ray marker on CT and the solid applicator models on CT and MR, as well as the percent dose difference for the prescription and inferior reference points, is shown in Table 2. The mean source position deviation for the segmented cylinders is 0.4 mm, 0.9 mm, and 0.6 mm for the CT, T1W, and T2W images, respectively. The average, absolute percent dose difference for the prescription and inferior reference points are 1.3% and 1.3% for the CT, 2.6% and 2.0% for the T1W, and 2.2% and 3.1%, respectively, for the T2W images. The mean source position deviation for the standard vaginal cylinders is 0.5 mm, 0.5 mm and 0.8 mm for the CT, T1W, and T2W images, respectively. The average absolute percent dose difference for prescription and inferior reference points are 2.5% and 2.7% for the CT, 2.1% and 2.3 % for the T1W, and 3.2% and 2.9% for the T2W images.

For the last dataset, the three‐patient cohort, a summary of the differences in central source positions based on the conventional digitization technique, as well as the percent dose difference for the bladder, rectum, prescription, and inferior reference point, is presented in Table 3. The mean source position deviation for all three patients is 0.9 mm, 1.2 mm, and 1.4 mm for the CT, T1W, and T2W images, respectively. The average absolute percent dose difference for the bladder, rectum, prescription, and inferior reference points are 2.9%, 2.6%, 3.0%, and 4.2% for the CT, 6.5%, 1.6%, 2.5%, and 4.7% for the T1W, and 6.0%, 7.4%, 2.6%, and 2.0%, respectively, for the T2W images.

**Table 2:** Magnitude of central source deviations and percent dose differences for a series of commercially available standard and segmented vaginal cylinders scanned in a water phantom. The cylinders were digitized with a commercial solid applicator model, as well as the conventional method of applicator reconstruction, based on an X‐ray marker in CT

		*Min (mm)*	*Max (mm)*	*Mean (mm)*	*SD (mm)*	*% Dose Difference Prescription Point*	*% Dose Difference Inferior Reference Point*
Segmented 2.0 cm	Solid CT vs. X‐ray marker	0.4	0.5	0.4	0.0	−2.2	−1.6
	Solid T1W vs. X‐ray marker	0.4	0.6	0.5	0.1	−2.9	−1.7
	Solid T2W vs. X‐ray marker	0.3	0.7	0.5	0.1	2.6	3.4
Segmented 2.6 cm	Solid CT vs. X‐ray marker	0.5	0.7	0.6	0.1	−0.3	0.8
	Solid T1W vs. X‐ray marker	0.9	1.1	1.0	0.0	1.1	3.0
	Solid T2W vs. X‐ray marker	0.5	0.5	0.5	0.0	2.8	3.7
Segmented 3.0 cm	Solid CT vs. X‐ray marker	0.3	0.5	0.4	0.1	2.2	2.9
	Solid T1W vs. X‐ray marker	0.5	0.9	0.6	0.1	0.5	2.6
	Solid T2W vs. X‐ray marker	0.4	0.7	0.5	0.1	2.5	3.7
Segmented 3.5 cm	Solid CT vs. X‐ray marker	0.2	0.4	0.3	0.1	−0.6	0.0
	Solid T1W vs. X‐ray marker	1.3	2.0	1.6	0.2	−5.8	0.6
	Solid T2W vs. X‐ray marker	0.9	1.0	1.0	0.1	−0.8	1.5
Standard 2.0 cm	Solid CT vs. X‐ray marker	0.2	0.3	0.3	0.0	−2.3	−2.4
	Solid T1W vs. X‐ray marker	0.2	0.3	0.3	0.0	−0.7	−0.2
	Solid T2W vs. X‐ray marker	0.5	0.7	0.6	0.0	2.3	3.7
Standard 2.3 cm	Solid CT vs. X‐ray marker	0.5	0.5	0.5	0.0	4.9	5.6
	Solid T1W vs. X‐ray marker	0.5	0.6	0.6	0.0	−0.7	0.5
	Solid T2W vs. X‐ray marker	0.5	1.0	0.7	0.1	5.0	1.5
Standard 2.6 cm	Solid CT vs. X‐ray marker	0.2	0.5	0.4	0.1	−0.8	0.2
	Solid T1W vs. X‐ray marker	0.4	0.5	0.5	0.0	−2.1	−1.8
	Solid T2W vs. X‐ray marker	0.5	0.7	0.6	0.0	−1.7	−2.2
Standard 3.0 cm	Solid CT vs. X‐ray marker	0.3	0.5	0.4	0.1	3.5	3.4
	Solid T1W vs. X‐ray marker	0.4	0.5	0.5	0.1	4.4	4.7
	Solid T2W vs. X‐ray marker	0.3	0.8	0.5	0.1	4.7	4.2
Standard 3.5 cm	Solid CT vs. X‐ray marker	0.6	0.8	0.7	0.0	1.0	2.1
	Solid T1W vs. X‐ray marker	0.8	1.0	0.9	0.1	2.8	4.2
	Solid T2W vs. X‐ray marker	1.5	1.7	1.6	0.1	−2.4	2.7

**Table 3:** Magnitude of source displacement of the central source position and percent dose difference at reference points when applicator reconstruction based on the solid applicator model is compared to reconstruction based on X‐ray markers for three different patients

		*Min (mm)*	*Max (mm)*	*Mean (mm)*	*Std. Dev. (mm)*	*%Dose Difference Prescription Point*	*%Dose Difference Inferior Reference Point*	*%Dose Difference Bladder*	*%Dose Difference Rectum*
Patient 1 Standard 2.6 cm	Solid CT vs. X‐ray marker	0.5	1.1	0.8	0.2	2.9	4.2	−4.8	3.0
	T1W vs. X‐ray marker	0.5	0.8	0.6	0.1	1.6	3.2	−4.0	2.9
	T2W vs. X‐ray marker	0.7	0.9	0.8	0.0	−3.4	−2.8	1.8	1.0
Patient 2 Standard 2.6 cm	Solid CT vs. X‐ray marker	1.2	1.3	1.2	0.0	5.3	7.8	−0.1	−4.5
	T1W vs. X‐ray marker	1.0	1.3	1.1	0.1	−0.2	2.9	−3.1	0.7
	T2W vs. X‐ray marker	1.6	2.5	2.0	0.3	−4.1	−1.6	11.3	−13.9
Patient 3 Standard 3.0 cm	Solid CT vs. X‐ray marker	0.5	0.8	0.7	0.1	0.7	−0.6	−3.7	−0.3
	T1W vs. X‐ray marker	1.6	1.9	1.7	0.1	5.9	8.0	−12.5	−1.1
	T2W vs. X‐ray marker	1.3	2.0	1.6	0.2	0.2	−1.8	−4.8	−7.3

## IV. DISCUSSION

Recommendations and guidelines for image‐guided brachytherapy for gynecological malignancies have mainly focused on the treatment of cervical cancers;^8^, ^9^, ^10^, ^11^, ^13^, ^14^, ^15^, ^16^, ^17^, ^18^, ^19^, ^20^, ^21^, ^22^ there are limited studies^23^, ^24^, ^25^, ^26^, ^27^ discussing image‐guided vaginal cylinder brachytherapy for post‐surgery endometrial carcinoma. CT is the most common imaging modality for 3D based image‐guided vaginal cylinder brachytherapy. It has been used to assess air gaps at the apex of the vagina,^(26)^ the effect of bladder filling,^25^, ^27^ to perform dose–volume histogram (DVH) analyses,^(24)^ and the effect of single versus multichannel cylinder in high‐dose‐rate brachytherapy of vaginal cuff.^23^, ^28^ However, with the increased accessibility of MR imaging and its superior soft‐tissue contrast, there has been an increase in its usage for brachytherapy treatment planning and treatment response evaluation. We have recently presented preliminary data from our institution suggesting that based on T2W MR images, the vaginal cuff is underdosed in over half of our patients due to limitations in the design of the standard vaginal cylinders and/or changes in surgical practices.^(29)^ Efforts of other groups at creating custom mold applicators constructed with 3D printers^30^, ^31^ further demonstrate the emerging need for more precise, tissue‐directed planning for vaginal brachytherapy.

In this study, we share our experience with the clinical implementation of MR‐guided brachytherapy, which allows for simulation and planning of vaginal cylinder brachytherapy patients based solely on MR imaging. Applicator reconstruction is an important part of this process. There have been several proposed methods for applicator reconstruction using MR images including the use of an MR compatible marker^32^, ^33^ and an applicator library.^13^, ^34^ Each of these methods have known limitations, such as the limited availability of commercial MR compatible markers, difficulties fabricating leak‐tight MR markers, and limits to the available applicators for which vendors have provided applicator models.

At our institution, we began investigating applicator reconstruction on MR images by using in‐house MR‐visible markers for both T1W and T2W MR images. However, as demonstrated in Fig. 2, we encountered difficulties maintaining a water‐tight seal of these markers. Several different techniques were employed to seal the catheters including a heat seal with and without hot glue, bone wax with cyanoacrylate, and Water Weld (JB Weld, Sulphur Springs, TX) with and without cyanoacrylate; however, none of these methods were successful. As such, our group discontinued the use of the in‐house markers for applicator reconstruction. We believe that a commercial solution would help mitigate this issue.

The main focus of this study was to evaluate applicator reconstruction on CT and MR images using solid applicator models available in a commercial treatment planning system. This was accomplished by retrospectively reviewing a 15‐patient cohort undergoing vaginal cylinder brachytherapy, as well as a series of phantom scans acquired with standard and segmented cylinders of a variety of sizes. Our measurements lead us to believe that the solid model technique is a reliable and reproducible alternative to our conventional technique for applicator reconstruction (based on X‐ray markers) of vaginal cylinders. A slightly larger source deviation was observed *in vivo* due to factors such as internal organ motion. The associated deviation was larger on MR images due largely in part to the longer scan times required for MR images (on average 250 s for 3D T1 MPRAGE scan, 720 s for 3D T2 Space scan, versus 10 s for a 1 mm slice thickness CT scan). The increase in scan time can result in degradation in the quality of the MR images such as artifacts and blurring due to organ and patient motion, which presents challenges during applicator reconstruction. As such, we have transitioned to the use of 3D T1W VIBE scans, which have a considerably shorter acquisition times (on the order of 90 s). Although antiperistalsis agents, such as glucagon, are commonly used for pelvic MR imaging to reduce image artifacts, especially for longer scan sequences,^35^, ^36^ due to the associated side effects of this medication (e.g., nausea and vomiting^(37)^) we have elected not to use this agent for brachytherapy patients.

In this study, applicator reconstruction has been performed for vaginal cylinders with solid applicator models on T1W and T2W MR images from a 3T MR scanner. Once validated, this method of applicator reconstruction can be applied to other applicators and images acquired from MR scanners with other field strengths. Applicator reconstruction strategies introduced in this study may not be directly transferrable to other treatment planning systems, but similar methods, such as applicator reconstruction based on user defined library plans, may prove to be useful when a reliable MR marker is not available. However, it is imperative that commissioning include both phantom and *in vivo* scans to validate the accuracy and reproducibility of applicator reconstruction prior to clinical implementation of MR‐based brachytherapy.

## V. CONCLUSIONS

The clinical implementation of MR‐guided vaginal brachytherapy in our department has been described. We investigated the use of commercial solid applicator models for applicator reconstruction. Based on this study, the solid applicator model provides a practical approach to vaginal cylinder applicator reconstruction for MR‐based brachytherapy planning. This will facilitate the transition to volume‐based planning which has the added advantage of improved soft‐tissue visualization and possible utilization of functional imaging.
